# Spasm of the Masseters, with Complications1Read before the Illinois State Veterinary Medical Association, February 21, 1900.

**Published:** 1900-03

**Authors:** J. D. Nighbert

**Affiliations:** Pittsfield, Ill.


### SPASM OF THE MASSETERS, WITH COMPLICATIONS.[1]

BY J. D. NIGHBERT, V.S.,
PITTSFIELD, ILL.

UNDER the above heading I shall call your attention to a few of the many cases of this disease or condition coming under my observation, not that I can give you the true etiology, but to add my mite to the very scant literature on the subject.

Generally seen in warm weather and in animals that have been at rest for a more or less lengthy period, and usually seen after vigorous or extended exercise. A majority of the cases coming under my observation were in animals that were taken up from pasture and put suddenly to work. I am inclined to think that this condition arises in a great many cases from some gastric disturbance depending on disturbed nutrition and the circulation of impure blood through the cranial structures.

After somewhat prolonged exercise a profuse perspiration is noticed, and if exercise is continued this will soon cease, and the

[1] Read before the Illinois State Veterinary Medical Association, February 21, 1900.

animal becomes dry; then will be noticed an inability or lagging in harness, and on being brought to a standstill there will be some uneasiness, with spasm of diaphragm and slight colicky pains, which soon pass away, followed by an inclination to wander around the enclosure or stall. At this stage may be noticed a slight turning up of the upper lip owing to spasm of the levators of the lip being in a tonic state; the masseters are soon in the same condition. While the temporals are also involved, they maintain a clonic condition throughout the attack.

Breathing accelerated; heart beats rapidly, but pulse at the jaw is almost imperceptible; temperature high, varying from 105° to 108°. After the initiatory stage the power of deglutition is lost. The duration of this condition is from a few to twenty-four hours.

Case I.—Aged roan mare, taken from pasture and put to work on a very warm day. About the middle of the afternoon she was noticed to be sweating profusely, and inclined to stagger, and, after resting a few minutes, refused to go when spoken to. She was taken out and I was sent for. Found patient as follows: sweating considerably, hurried breathing, nostrils dilated, heart beating rapidly, pulse almost imperceptible at the jaw, well-marked spasm of diaphragm, mare wandering around, gait stilty, masseters in tonic spasm, temporals clonic, upper lip turned up and levators in tonic spasm; temperature 106°; unable to drink; would put her nose almost up to the eyes in the bucket in a vain attempt to get a swallow of water.

*Treatment.* A liberal abstraction of blood from the jugular, a hypodermic of atropine, and had the patient taken to the shade; cold water continually applied to poll; left one ounce of potassium nitrate to be given in drinking-water as soon as the patient could swallow; told owner I would call again the next morning. Mare became easy and quiet about midnight, and was left to herself for the rest of the night. When I called the next morning I found the patient somewhat improved and very anxious to drink, but was still unable to swallow. I inserted a tube through the nostril into the œsophagus, attached the hose of the syringe, and gave her about two gallons of water in which one-half ounce of potassium nitrate had been dissolved. This acted like magic, and in a very few minutes she was able to swallow and was anxious for feed. Ordered her kept in the shade and fed lightly.

Case II.—Bay pony, taken up from pasture and driven into town, a distance of about nine miles. Symptoms similar to Case I., with well-marked emprosthotonus. Would place her head almost to the ground between the front legs, seemingly to attempt to turn a somersault. Treatment similar to the above, but used tube twice about three hours apart. Symptoms soon subsided. Case did well.

CASE III.—Bay mare, with colt by her side, had been kept in small paddock. Served early in morning of ninth day and put in box-stall. In the course of an hour or so the owner noticed a slight spasm of the diaphragm, but as the mare appeared to be busy eating hay from the manger he thought there was nothing serious, and left her until evening, when her condition was worse. I was called. Found the patient with well-marked spasm of the diaphragm, masseters, etc., and a mouthful of semi-masticated hay. Treatment the same as the previous cases. No improvement. Mare died at 5 A.M.

